# Intelligent Personalized Exercise Prescription Based on an eHealth Promotion System to Improve Health Outcomes of Middle-Aged and Older Adult Community Dwellers: Pretest–Posttest Study

**DOI:** 10.2196/28221

**Published:** 2021-05-24

**Authors:** Ting Sun, Yang Xu, Hui Xie, Zuchang Ma, Yu Wang

**Affiliations:** 1 School of Nursing Bengbu Medical College Bengbu China; 2 Anhui Province Key Laboratory of Medical Physics and Technology Institute of Intelligent Machines Hefei Institutes of Physical Sciences, Chinese Academy of Sciences Hefei China

**Keywords:** exercise prescription, cardiovascular function, body composition, bone mineral density, physical fitness

## Abstract

**Background:**

A scientific, personalized, and quantitative exercise prescription that has the potential to be an important therapeutic agent for all ages in the prevention of chronic disease is highly recommended. However, it is often poorly implemented, as clinicians lack the necessary knowledge and skills while participants have low adherence due to design defects (eg, prescriptions fail to take individual willingness, the appeal of exercise, and complex physical conditions into account). Intelligent personalized prescription is thus worth exploring.

**Objective:**

The aim of this study was to investigate whether a year-long cloud platform–based and intelligent personalized exercise prescription intervention could improve Chinese middle-aged and older adult community dwellers’ health outcomes.

**Methods:**

A total of 177 participants (aged 52-85 years; mean 67.93, SD 7.05) were recruited from 2 Chinese community health service centers in Anhui Province, China. The exercise intervention was delivered over 12 months with a single-group pretest–posttest design. After being assessed in terms of physical activity, health-related lifestyle, history of chronic diseases and drug use, family history of disease and cardiovascular function, body composition, bone mineral density, and physical fitness through an eHealth promotion system, participants with relative contraindications for exercise were personally prescribed the health care exercise mode by an intelligent system, while those without relative contraindication and who had a regular exercise habit were prescribed the scientific fitness mode. Paired *t* tests were used for the analysis.

**Results:**

A total of 97 participants were classified into the health care mode, and the remaining 80 participants were assigned to the scientific fitness mode. Significant changes in heart rate (mean difference [MD] 2.97; 95% CI 1.1-4.84; *P*=.002), subendocardial viability ratio (MD –0.13; CI: –1.19 to –0.63; *P*<.001), weight (MD 0.99; CI 0.29-1.69; *P*=.006), BMI (MD 0.38; CI 0.11-0.64; *P*=.006), body fat rate (MD 0.88; CI 0.24-1.51; *P*=.007), fat mass (MD 0.92; CI 0.33-1.53; *P*=.003), and brachial-ankle pulse wave velocity (MD: –0.72; CI –1.17 to –0.27; *P*=.002) were observed among participants with the health care mode exercise prescriptions at the 12-month postintervention versus the baseline assessment, while no changes in systolic blood pressure, diastolic blood pressure, muscle mass, bone mineral density, *t* value, *z* value, balance, or ability were discerned. The results showed a functional decline in the physical fitness of both groups, including in handgrip strength (healthcare mode: MD 4.41; scientific fitness mode: MD 3.11), vital capacity (healthcare mode: MD 261.99; scientific fitness mode: MD 250.78), and agility (healthcare mode MD=–0.35; scientific fitness mode: MD=–0.39) with all *P* values <.001, except handgrip strength in the scientific fitness mode (*P*=.002). There were no significant differences in other parameters among participants with scientific fitness mode exercise prescriptions.

**Conclusions:**

The observations suggest that our exercise prescription intervention program might promote certain health outcomes in Chinese middle-aged and older adult community dwellers, yet we are unable to recommend such a program given the existing limitations. Future randomized controlled trials with diverse samples are warranted to confirm our findings.

## Introduction

Physical activity is defined as any bodily movement produced by skeletal muscle [1]. Exercise is one form of delivery for physical activity and has been defined as a structured activity to maintain essential physiological systems, such as the skeletal muscular and metabolic systems [2], and to improve physical function and quality of life [1,3-5]. Resistance training is considered a very effective method for the development of skeletal muscle [6], and aerobic exercise training performed at an appropriate level of intensity has beneﬁcial effects on cardiopulmonary function [7]. As middle-aged and older adults experience functional decline and often suffer from one or more noncommunicable chronic diseases (NCDs), many people have become aware of the health benefits of regular exercise. Consistent evidence confirms the benefits of exercise: prevention of injurious falls, sarcopenia, cognitive decline, and frailty [3,8,9]; a reduction of morbidity (eg, coronary heart disease, type 2 diabetes) [2,3,10,11]; and at least a 20%-30% reduction for more than 25 chronic symptoms (eg, depression or hypertension,) and 10% reduction in premature mortality [10,12]. Furthermore, a lack of exercise not only leads to an increase in NCDs, but also increases medical costs and the economic burden on the state and individuals [4], which is estimated to be at about US $68 billion worldwide annually [10]. The benefits of exercise for everyone are obvious. Even for middle-aged and older adults with NCDs, exercise is one of the best medicines, being cheap, accessible, and providing a complex, whole-body impact with very few side effects [13]. Therefore, exercise training is a cornerstone in the management of NCDs [14].

Although the value of regular exercise is well known, many people still do not include exercise in their daily routines [15]. A global pandemic of physical inactivity has been described [10]. For example, 50% of American adults and 49% of New Zealand adults are nonactive or insufficiently active [2,12]. The situation for older people may be worse as a result of complex physical conditions [16]. Older individuals may struggle to exercise because of pain or other symptoms [17].

Exercise prescription is cost-effective and can increase physical activity by 10% in relatively inactive patients [1]. It is recommended and defined by the World Health Organization (WHO) as follows: an exercise prescription given by physicians to help patients engage in physical exercise, according to the medical examination data (including exercise and physical tests) and based on the patient’s specific health, physical strength, and cardiovascular function status. The type of exercise, exercise intensity, exercise time, and exercise frequency are specified in the prescription, and any associated precautions when undertaking the exercise are detailed [18]. The American College of Sports Medicine (ACSM) introduced an exercise prescription guideline in 1990 to guide the design of exercise prescriptions for the general population and people with chronic diseases [19].

Exercise prescription is highly recommended by the current guidelines on the prevention and management of chronic disease, but its implementation remains poor [20]. Many clinicians—especially primary care staff [1]—experience difficulties in prescribing exercise in the presence of different concomitant chronic diseases and risk factors within the same patient because of a lack of expert knowledge and skills [21,22]. Although the scientific effectiveness of exercise prescriptions is supported by a substantial amount of experimental data, studies have also demonstrated that adherence to this “scientific” and “best health–benefit” exercise prescription is not high [23,24]. This is likely because the prescription design does not take into account factors such as individual willingness to exercise, the appeal of exercise, and the conditions under which exercise occurs. In short, personalized exercise prescription is not simple and comprises many challenges, such as time constraints, complex comorbidities, perceived lack of patient engagement, and a lack of physician training or education on the particulars of physical activity [1,13]. To provide an effective and safe exercise program for patients with multiple comorbidities, an advanced intelligent exercise prescription that can calculate the complex physical condition of a patient and combine this with the patient’s exercise preferences to give the best prescription, is required [25]

The purpose of this year-long program was to verify the effectiveness of intelligent personalized exercise prescriptions for middle-aged and older adult community dwellers and support the next research stage: exercise prescription using the smartphone with intervention and procedural monitoring through wearable devices. Ultimately, we envision a new protocol that includes 3 characteristics: (1) an individual assessment of needs, motivation, habits, preferences, and barriers; (2) valid behavior change approaches; and (3) proper follow-up, self-monitoring, and social support.

## Methods

### Design

This study included a single-group pretest–posttest design with blind pre- and postoutcome assessment and the collector of participant information being unaware of the purpose of the experiment.

### Study Sample and Recruitment

Between January 2019 and November 2019, middle-aged and older adult community-dwelling individuals were recruited via 2 community health service centers in Bengbu in the Anhui Province of China. They were invited by center staff through telephone or verbal invitation, and those interested were asked to contact staff members for further information. Eligible participants were then scheduled to be pre-examined one by one. When they finished the entire year-long intervention program, the time of the postintervention evaluations was booked, and they were reassessed one by one until the last participant finished in November 2020. There were 3 modes available for the intervention, but because of difficulties in meeting requirements for the “exercise habit formation” mode, we only recruited participants for the “health care” and “scientific fitness mode” groups. The participants were required to be more than 50 years of age, be community dwellers, have no regular exercise habits (total number of physical activities of various intensity per week ≥3 times; total physical activity time per week ≥90 minutes) for inclusion into the health care mode group, and have regular exercise habits for inclusion into the scientific fitness mode group. Participants were excluded if they had serious cardiovascular or cerebrovascular diseases; lung or kidney diseases or related complications, according to their self-reported hospitalization experience; severe diabetes or related complications, such as fundus lesion, peripheral neuropathy, diabetic foot, or renal dysfunction; a fasting blood glucose ≥13.3 mmol/L and urine ketone positive, or postprandial blood glucose ≥19.4 mmol/, with resting blood pressure ≥180/110 mmHg; or severe cognitive impairment and diagnosed mental disorders, such as moderate to severe depression, schizophrenia, and mania.

### Ethical Considerations

In accordance with ethical guidelines, the participants were fully informed about the positive effects of exercise prescription and the details of the procedures. All participants provided written informed consent to participate in this study and agreed to their data being used. The study protocol was approved by the Ethics Committee of Bengbu Medical College (Anhui, China; no. 2018045).

### Sample Size

We calculated the sample size of the mean difference (MD) of the before and after comparison of each index, as reported in similar literature [26], using MedSci sample size tools with α=.05 and 1-β=.8 resulting in a sample range of 11-67. A sample size of 70 was decided upon for both groups.

### Intervention

**Figure 1:**
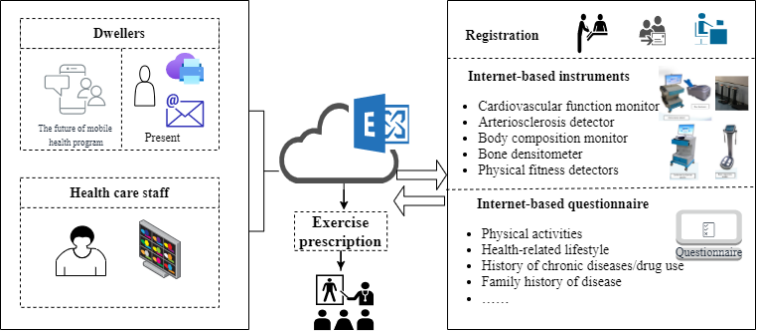
Components of the eHealth promotion system.

This intelligent personalized exercise prescription program (IPEPP) is a preliminary experiment for a mobile health intervention, based on an eHealth promotion system (Figure 1). At present, it includes 4 elements: (1) registration system (typically participants are invited to participate in the program by a family-contracted physician); (2) cloud platform (all data are stored and calculated in the cloud platform, through which community health care staff and researchers can monitor an individual’s data online); (3) internet-based instruments (including cardiovascular function monitor, arteriosclerosis detector, body composition monitor, bone densitometer, and physical fitness detectors used to assess cardiovascular function, body composition, bone mineral density, and physical fitness of participants before and after intervention); (4) an internet-based questionnaire to collect data about physical activity, health-related lifestyle, history of chronic diseases or drug use, and family history of disease. In the future, a mobile health app currently under development will be combined with wearable devices for monitoring the process of exercise to increase the adherence of intervention based on theories of health behavior change.

The IPEPP comprised 4 processes: (1) assessment (using internet-based instruments and an internet-based questionnaire to collect integrated information and analyze the situation of the individuals to support decision-making about individualized exercise prescription), (2) exercise prescription generation (based on the assessment of the integrated information and the individual’s exercise habits, 3 types of personalized exercise prescription are generated: a health care mode, an exercise habit-formation mode, and a scientific fitness mode), (3) execution and supervision (health care staff print exercise prescriptions or send them to participants by email, explain the instructions, precautions, and content of exercise and behavior modification, and follow up with participants every 2 weeks by telephone), and (4) evaluation of exercising effects (cardiovascular function, body composition, bone mineral density, and physical fitness).

### Assessment

**Figure 2:**
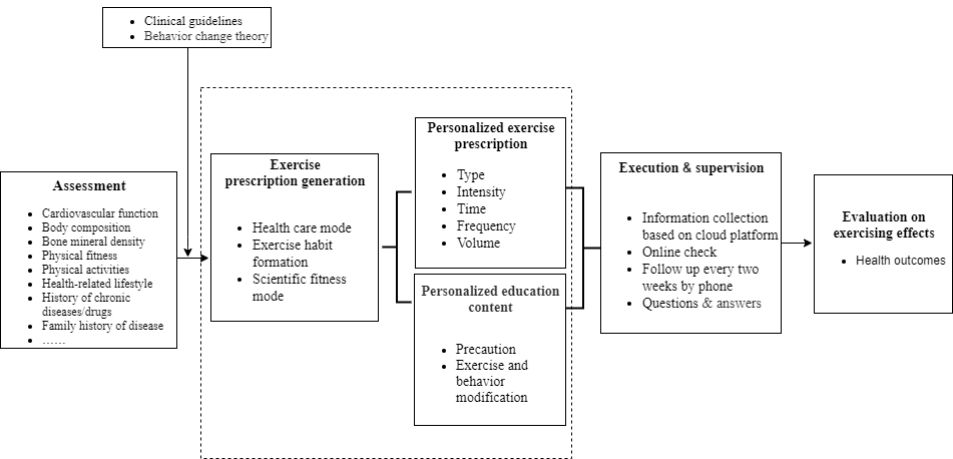
The assessment, exercise prescription generation, execution and supervision, and evaluation of exercising effects of the intelligent personalized exercise prescription program intervention.

**Figure 3:**
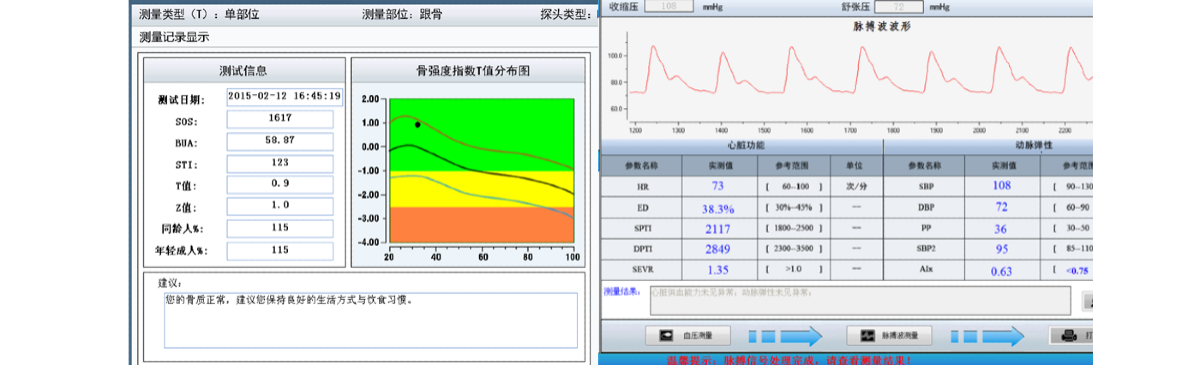
Examples of screenshots of the examination reports of bone mineral density and cardiovascular function.

According to the ACSM’s guidelines for exercise testing and prescription, pre-exercise screening is indispensable [19]. The generation of our exercise prescription was based on the assessment of baseline data. It consisted of information collected by an internet-based questionnaire system, including the individual’s current level of physical activity, history of chronic diseases (eg, cardiovascular and cerebrovascular diseases, diabetes and hypertension) and drug use (eg, types, dosage, and times), symptoms of the disease (eg, exercise dysfunction, hypoglycemia, and hypotension symptoms), health-related lifestyle (eg, eating, smoking and drinking habits), and family history of disease (Figure 2; other details are published elsewhere [27]); and information collected by internet-based instruments, which included physiological parameters (cardiovascular function, body composition, bone mineral density, and physical fitness) as obtained from a body composition monitor (BX-BCA-100, Institute of Intelligent Machines), a cardiovascular function monitor (BX-CFTI-100, Institute of Intelligent Machines), an arteriosclerosis detector (BX-AS-100, Institute of Intelligent Machines), a bone densitometer (BX-BDI-500A, Institute of Intelligent Machines), and physical fitness detectors, including a handgrip strength meter (TSN100-WL, Physical Fitness Sports Technology Company), a reaction time meter (TSN100-FY, Physical Fitness Sports Technology Company), a 1-leg stand meter with closed eyes (TSN100-ZL, Physical Fitness Sports Technology Company), a spirometer (TSN100-FH, Physical Fitness Sports Technology Company), and a flexion measurement instrument of sitting position (TSN100-TQ, Physical Fitness Sports Technology Company). Figure 3 displays examples of screenshots showing examination results of the bone densitometer and cardiovascular function monitor. The details of the assessment procedure are shown in Multimedia Appendix 1, and other related information has been published elsewhere[27].

### Exercise Prescription Generation

**Figure 4:**
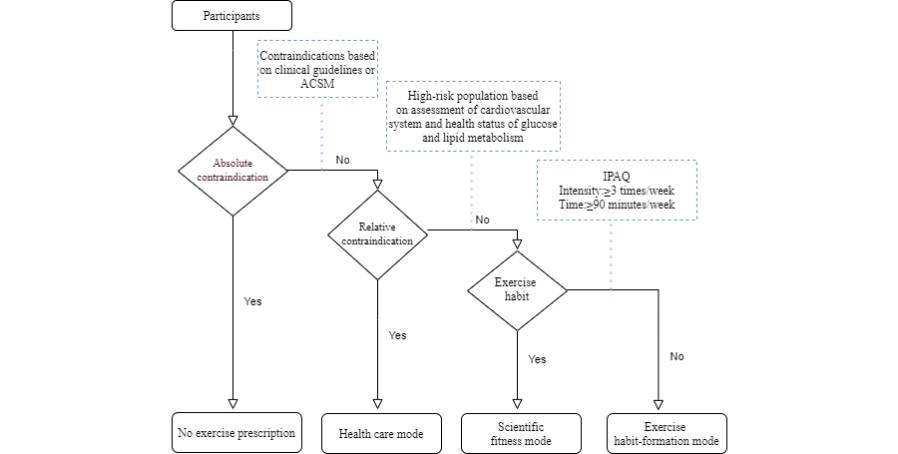
Algorithms for 3 modes of exercise prescription. ACSM: The American College of Sports Medicine. IPAQ: International Physical Activity Questionnaire.

All exercise prescription recommendations were based on clinical guidelines and the ACSM. Participants for whom exercise was absolutely contraindicated were excluded, while those with relative contraindications were given the health care mode as their prescription. Relative contraindications included the following: suspected symptoms or target organ damage, as indicated by subendocardial viability ratio (SEVR) ≤0.9; ankle/brachial systolic pressure index (ABI) ≥1.4 or 0.6 ≥ ABI >0, brachial-ankle pulse wave velocity (baPWV) ≥21.1 m/s; central arterial pressure ≥145 mmHg; and abnormal blood pressure, as indicated by systolic blood pressure (SBP) ≥160 mmHg or diastolic blood pressure (DBP) ≥100 mmHg, and SBP <90 mmHg or DBP <60 mmHg with hypotensive symptoms. After being confirming of having no relative contraindications, participants with or without adequate and regular physical activities were given the scientific fitness mode or exercise habit-formation mode exercise prescriptions (Figure 4).

**Figure 5:**
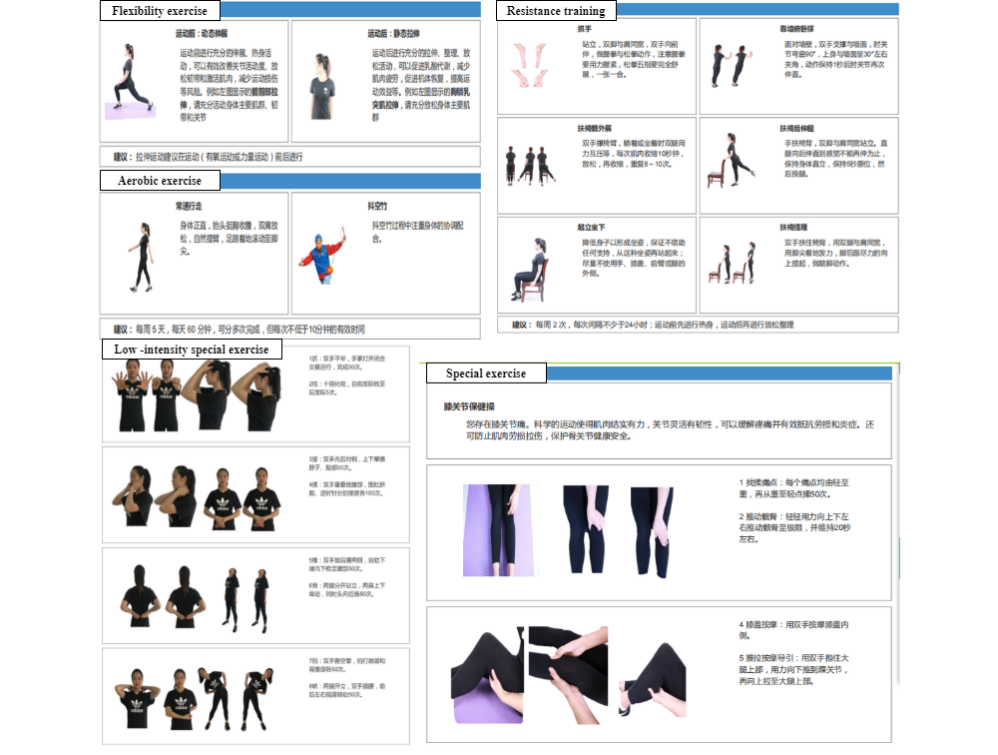
Examples of exercise instructions.

The system automatically generated 3 kinds of exercise prescriptions with pictures of the recommended exercise type (Figure 5) and notes of exercise precautions corresponding with the screened risks and incorrect exercise-related health behaviors. The health care mode recommended low-intensity aerobic gymnastics and other simple types of low-intensity exercise, including basic self-massage exercises (randomly drawn from the system). Compared with the health care mode, the exercise habit-formation mode recommended an unstructured prescription to cultivate individuals’ exercise consciousness. It encouraged individuals to choose their exercise type and intensity according to their subjective feelings, and exercises were recommended with low requirements for skills or physical fitness, such as walking, leisure cycling, and slow dances. It is generally recommended that a single effective exercise time should not be less than 10 minutes and that the frequency of exercise should not be less than twice a week. The scientific fitness mode recommended a structured prescription following the frequency, intensity, type, time, volume, and progression principle, which includes aerobic exercise and resistance training (Table 1). Examples of exercise prescriptions and algorithms for various exercise categories are presented in Multimedia Appendices 2 and 3.

**Table 1:** Matrix of links between the categories of personalized exercise prescriptions and exercise types.

Prescription type	Aerobic exercise	Resistance training	Flexibility exercise	Special exercise^a^	Low-intensity aerobic gymnastics
Health care mode	√^b^				√
Exercise habit-formation mode	√		√	√	
Scientific fitness mode	√	√	√	√	

^a^Special exercise is recommended by prescription when a related health problem is detected in the exercise habit-formation mode and scientific fitness mode.

^b^√: The type of exercise recommended by prescription.

The exercise habit-formation mode and scientific fitness mode include recommendations for flexibility exercises before and after either aerobic exercise or resistance training (Figure 5). Special exercises were recommended for any currently detected health problems (eg, knee pain, neck discomfort), and the system recommended a suitable set of health care exercises from the special sports library as guidance. Only when relevant problems were detected in the participants during the assessment process would these special exercises be recommended (Table 1).

### Execution, Supervision, and Evaluation of Exercising Effects

**Figure 6:**
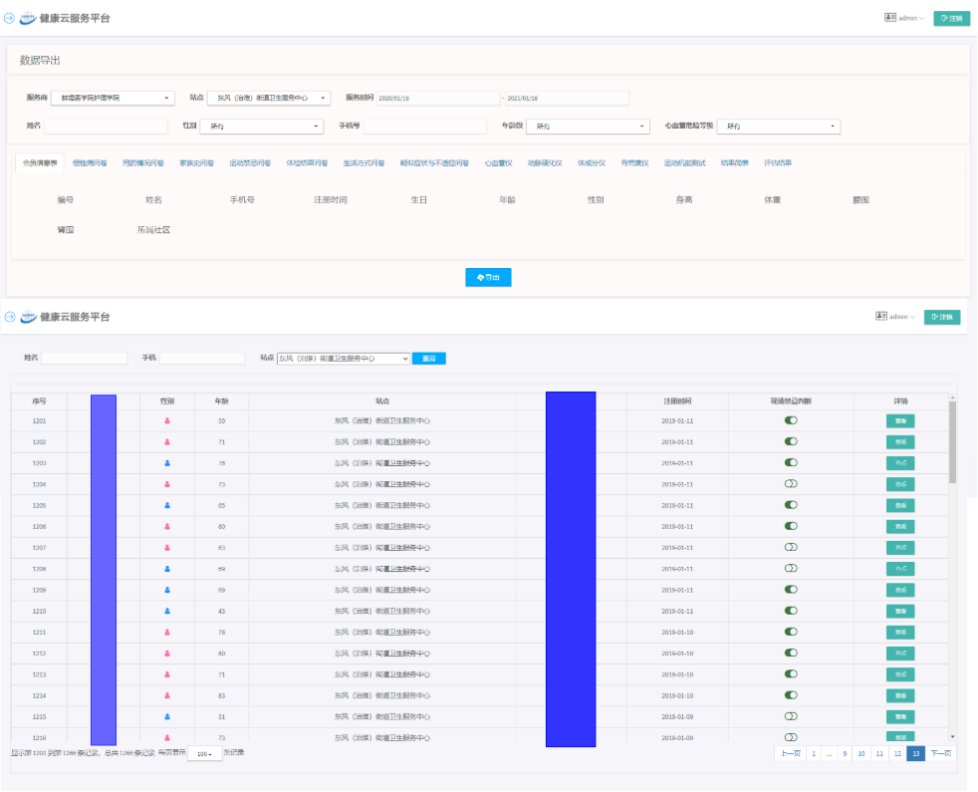
Website with cloud platform screenshot.

Participants were given their exercise prescription in the community health center after a detailed explanation, which culminated in them taking away printed color papers or receiving emails. Researchers visited the cloud platform online to find each individual’s information (Figure 6), and they followed up participants every 2 weeks by telephone to remind them to exercise according to their prescription (keeping the same prescription for a year) and to help them solve any problems encountered.

After 12 months, the participants were reevaluated to check if there were any changes in the following health outcomes: cardiovascular function, body composition, bone mineral density, and physical fitness. Participants were asked to report whether they changed their medication use or related lifestyle (eg, diet change).

### Statistical Analyses

Data were analyzed using SPSS 23.0 software (IBM Corp). Continuous variables are expressed as mean and SD. Paired *t* tests for independent samples and Pearson chi-squared test were used to assess the significance of differences in baseline characteristics between the health care mode and scientific fitness mode. Paired *t* tests were also used to analyze the difference in health outcomes before and after intervention. A *P* value <0.05 was considered statistically significant.

## Results

### Participants

A total of 232 participants agreed to participate in the study. Of this number, 27 participants were excluded due to not meeting inclusion criteria, and 28 participants did not complete the entire program including those who changed medication. These 28 participants were excluded due to inadequate adherence, an inability to finish the postassessment, changes in health status unrelated to the study and thus cessation of the intervention, withdrawal, or loss of contact. Overall, 177 participants (105 women and 72 men; mean age 67.93 years, SD 7.05 years) were included in the final analysis, with 97 in the health care mode group and 80 in the scientific fitness mode group. All participants reported they did not change their related lifestyle.

### Baseline Data

Baseline characteristics are presented in Table 2. Epidemiological data of the 2 groups showed no difference except for gender, but participants with an exercise prescription in the health care mode had a poorer baseline condition for heart rate, SEVR, baPWV, body fat rate, fat mass, and vital capacity compared with those in the scientific fitness mode group. Other baseline characteristics did not differ between participants allocated to the 2 exercise prescriptions.

**Table 2:** Baseline data for health care mode versus the scientific fitness mode.

Characteristic			Health care mode (n=97)		Scientific fitness mode (n=80)		*P* value
**Epidemiological data**							
	Male gender, n (%)	30 (30.9)		42 (52.5)		.003	
	Age (years), mean (SD)	67.76 (6.67)		68.09 (7.42)		.76	
	Weight (kg), mean (SD)	68.18 (11.55)		68.06 (11.8)		.95	
	Cigarette consumption, (cigarettes/lifetime), mean (SD)	32022.16 (9934.85)		45579.37 (11378.80)		.37	
	Alcohol consumption (g/day), mean (SD)	5.97 (15.97)		5.63 (17.26)		.89	
	Sedentary time (min/day), mean (SD)	74.62 (86.04)		83.42 (85.35)		.51	
	Diabetes, n (%)	27 (27.8)		18 (22.5)		.27	
	Hypertension, n (%)	56 (57.7)		36 (45)		.06	
	Cardiovascular diseases, n (%)	18 (18.6)		15 (18.8)		.56	
**Cardiovascular function, mean (SD)**							
	SBP^a^ (mmHg)	130.22 (16.06)		125.96 (14.66)		.72	
	DBP^b^ (mmHg)	73.20 (9.10)		74.64 (8.73)		.29	
	Heart rate (bp/min)	71.66 (10.21)		67.77 (8.23)		.007	
	SEVR^c^	1.07 (0.19)		1.19 (0.20)		<.001	
	baPWV^d^ (m/s)	17.31 (3.19)		15.70 (2.70)		<.001	
**Body composition, mean (SD)**							
	Weight (kg)	68.18 (11.55)		68.06 (11.80)		.95	
	BMI (kg/m^2^)	25.82 (3.99)		25.17 (3.64)		.26	
	Body-fat rate (%)	30.50 (9.09)		27.03 (8.44)		.01	
	Fat free mass (kg)	47.18 (8.21)		49.56 (9.04)		.07	
	Muscle mass (kg)	44.57 (7.90)		46.90 (8.70)		.06	
	Fat mass (kg)	21.11 (8.67)		18.68 (7.28)		.046	
**Bone mineral density, mean (SD)**							
	STI^e^	84.41 (17.66)		90.63 (23.59)		.07	
	*t* value^f^	–1.13 (0.93)		–0.82 (1.24)		.08	
	*z* value^g^	0.46 (1.41)		0.94 (1.88)		.08	
**Physical fitness, mean (SD)**							
	Handgrip strength (kg)	25.01 (7.38)		26.65 (8.46)		.17	
	Vital capacity (ml)	1776.29 (579.87)		1947.74 (469.06)		.03	
	Agility (s)	0.89 (0.56)		0.76 (0.29)		.07	
	Balance ability (s)	4.78 (6.13)		5.29 (7.56)		.63	
	Flexibility (cm)	7.44 (6.73)		8.21 (6.40)		.44	

^a^SBP: systolic blood pressure.

^b^DBP: diastolic blood pressure.

^c^SEVR: subendocardial viability ratio.

^d^baPWV: brachial-ankle pulse wave velocity.

^e^STI: stiffness index.

^f^Used to evaluate the absolute risk of fracture.

^g^Primarily used to assess the relative risk of fracture for comparison between peers.

### Treatment Effect

Results of the health care mode group at baseline and posttest on health outcomes are shown in Table 3. Some domains of cardiovascular function and body composition, including heart rate, SEVR, weight, BMI, body fat rate, and fat mass, showed significant improvement. Compared with the scientific fitness mode group (Multimedia Appendix 4), no significant changes were observed in these parameters in the health care mode group. Noticeably, baPWV (MD –0.72; CI –1.17 to –0.27; *P*=.002) changed from 17.31 m/s to 18.03 m/s during the intervention periods in the health care mode participants, while no change was discerned in those following the scientific fitness mode.

For bone mineral density, no significant improvements were shown in either the health care (*P*=.682) or scientific fitness mode groups (*P*=.55). The results showed functional decline in the physical fitness of both groups, including handgrip strength (healthcare mode MD 4.41; scientific fitness mode: MD 3.11), vital capacity (healthcare mode: MD 261.99; scientific fitness mode: MD 250.78), and agility (healthcare mode: MD –0.35; scientific fitness mode: MD –0.39) with all *P* values <.001 except that of handgrip strength in the scientific fitness mode (*P*=.002).

**Table 3:** Changes of health outcomes in healthcare mode (N=97).

Characteristic		Pretest, mean (SD)	Posttest, mean (SD)	Difference, mean (95% CI)	*P* value
**Cardiovascular function**					
	SBP^a^ (mmHg)	130.44 (15.99)	132.41 (14.96)	–1.97 (–4.68 to 0.75)	.15
	DBP^b^ (mmHg)	73.29 (9.10)	74.54 (9.23)	–1.25 (–2.92 to 0.42)	.14
	Heart rate, (bp/min)	72.90 (10.45)	69.93 (8.53)	2.97 (1.1 to 4.84)	.002
	SEVR^c^	1.07 (0.20)	1.19 (0.28)	–0.13 (–0.19 to –0.06)	<.001
	baPWV^d^ (m/s)	17.31 (3.19)	18.03 (2.92)	–0.72 (–1.17 to –0.27)	.002
**Body composition**					
	Weight (kg)	68.18 (11.55)	67.18 (10.83)	0.99 (0.29 to 1.69)	.006
	BMI (kg/m^2^)	25.85 (3.99)	25.45 (3.65)	0.38 (0.11 to 0.64)	.006
	Body fat rate (%)	30.39 (9.13)	29.51 (8.88)	0.88 (0.24 to 1.51)	.007
	Fat-free mass (kg)	47.32 (8.22)	47.11 (8.14)	0.21 (–0.16 to 0.58)	.26
	Muscle mass (kg)	44.70 (7.92)	44.52 (7.84)	0.19 (–0.15 to 0.52)	.28
	Fat mass (kg)	21.15 (8.66)	20.15 (8.00)	0.92 (0.33 to 1.53)	.003
**Bone mineral density**					
	STI^e^	84.41 (17.66)	83.41 (19.56)	1.00 (–3.84 to 5.84)	.68
	*t* value^f^	–1.13 (0.93)	–1.19 (1.04)	0.53 (–0.21 to 0.31)	.69
	*z* value^g^	0.46 (1.41)	0.41 (1.53)	0.54 (–0.35 to 0.45)	.79
**Physical fitness**					
	Handgrip strength (kg)	25.01 (7.38)	20.60 (8.14)	4.41 (2.96, 5.86)	<.001
	Vital capacity (ml)	1776.29 (579.87)	1514.00 (568.43)	261.99 (143.84 to 380.14)	<.001
	Agility (s)	0.89 (0.56)	1.23 (0.80)	–0.35 (–0.53 to –0.17)	<.001
	Balance ability (s)	4.78 (6.13)	6.46 (8.64)	–1.68 (–3.54 to 0.17)	.08
	Flexibility (cm)	7.44 (6.73)	8.53 (7.15)	–1.08 (–2.68 to 0.51)	.18

^a^SBP: systolic blood pressure.

^b^DBP: diastolic blood pressure.

^c^SEVR: subendocardial viability ratio.

^d^baPWV: brachial-ankle pulse wave velocity.

^e^STI: stiffness index.

^f^Used to evaluate the absolute risk of fracture.

^g^Primarily used to assess the relative risk of fracture for comparison between peers.

## Discussion

We found a few improvements in cardiovascular function and body composition of participants with exercise-related contraindications but without good exercise habits. However, for participants already in the habit of exercising, there were no changes in these 2 domains.

Cardiovascular function and body composition improvements were important outcomes in our study. For cardiovascular function, we detected that heart rate and SEVR were signiﬁcantly improved following a 12-month unstructured exercise prescription intervention among middle-aged and older adult dwellers with related exercise contraindications. This is consistent with previous studies, in which exercise improved cardiovascular and arterial function [5] for healthy populations, and even sedentary individuals aged more than 50 years gained the benefit of improved maximal oxygen consumption after exercise [28]. Few studies have reported on improvement in SEVR among older adults, but Huang et al [29] found an augmentation in SEVR after a 6-week exercise intervention in obese adolescents. We suspect our low-intensity, unstructured exercise prescription with associated precautions and tips was a major contributor to adherence of participants who have doubts about how to exercise because of their disease symptoms. Our recommended low-intensity special aerobic gymnastics with instruction through pictures gave participants guidelines, and the unstructured prescription provided tools and cues for participants to take safety into consideration while choosing the physical activity they prefer. For body composition, those participants prescribed the health care mode changed their weight, BMI, body fat rate, and fat mass significantly, but their muscle mass was unchanged. Unsurprisingly, this is in line with results of other interventions with or without calory restriction, implemented in a variety of populations [25-30]. Most of these were short-term intervention programs (6 to 12 weeks), and evidence of long-term effectiveness of exercise intervention in middle-aged and older adults is insufficient. It can therefore be concluded that even low-intensity, aerobic exercise and unstructured physical activity is beneficial in the long-term for middle-aged and older adults without exercise habits. Essentially, starting to exercise is the most important thing.

Corresponding parameters of cardiovascular function and body composition in the scientific fitness mode group showed no statistically significant changes. We expected that the structured and combined exercise of aerobic and resistance training would improve cardiovascular function and body composition in participants with exercise habits. However, a possible ceiling effect might have prevented further improvements in functioning, even though these participants had completed more scientific exercise [17]. Theresa et al [30] reported that individuals with more “favorable” values at baseline (eg, lower submaximal heart rate) may potentially show a low subsequent training response if a “ceiling effect” limits further improvement in that parameter. Similar research showed low exercise doses can effectively reduce cardiovascular disease or cardiovascular risk factor prevalence, but higher exercise doses do not yield additional benefits [31].

Our present work showed an increase in baPWV for the health care mode group and unchanged results for the scientific fitness mode group. These changes differed from those reported in previous studies [32-35]. However, studies included in meta-analyses investigating the effects of exercise on arterial stiffness also report positive evidence [36-38] for aerobic exercise, with patients with isolated systolic hypertension being the exception. Resistance exercise has different effects on arterial stiffness depending on type and intensity, and there seem to be no unfavorable effects on arterial stiffness if the training is of low intensity or performed in a slow concentric manner or in the lower limbs of healthy individuals [37]. Furthermore, combined training has no significant effects on arterial stiffness [36]. In our study, more than half of the participants in with healthcare mode (56/97, 58%) and nearly half of the participants in the scientific fitness mode(36/80, 45%) were diagnosed with hypertension, and those in the health care mode group were given a low-intensity aerobic exercise prescription. This partly explains the lack of improvement in baPWV, as blood pressure is affected by many confounding factors (eg, medication). Another explanation might be age. Aging reduces arterial elasticity and has been suggested to be the main precursor of arterial stiffness in different populations [39,40], with this change being more significant in older adults. Noticeably, in our present work, arterial elasticity became worse in participants engaging in aerobic exercise only, but there was no significant difference in baPWV among community dwellers engaging in both aerobic exercise and resistance training. Given the undifferentiated baseline data, it may be that the potential effectiveness of this intelligent, personalized, structured exercise prescription intervention has been demonstrated. However, additional well-designed randomized controlled trials are needed before any ﬁnal recommendations can be formulated.

This 12-month intervention study showed no difference before and after intervention in bone mineral density for either group. Although some previous trials have reported the effectiveness of intervention [41], there is currently insufﬁcient evidence to recommend exercise for improving bone mineral density [42]. One meta-analysis reported that the positive results were small, nonsigniﬁcant, and with a large and statistically signiﬁcant amount of heterogeneity. There were also some consistent results detecting no significant changes in breast cancer survivors [43], or overweight and obese older adults [44].

Evidence does consistently suggest that exercise leads to signiﬁcant improvements in physical ﬁtness, increased ﬂexibility, agility, and strength [32,41,45-48]. However, in our present work, physical fitness showed a decline in both exercise prescription groups. The inclusion of older participants may partly explain this. Reduction in physical fitness, including reduction of muscle strength in both the upper and lower limbs, and changes in flexibility, agility, and endurance, were equal for both men and women and was likely due to the aging process, which was discussed in a previous study [49].

No adverse events were reported for any of the interventions. Although our prescription included step-by-step instructions with pictures and participants could call community health center staff to report any uncomfortable conditions during exercise, there are safety aspects worth considering that should be more strictly monitored. We should strengthen the monitoring of exercises so that harmful execution of exercises can be quickly noted and addressed.

There were several limitations to this research. First, this study was conducted in 1 geographic location, which limits the generalizability of observations and hinders the ability to identify population differences. Second, we only studied a single group of middle-aged and older adults and did not include a control group, impeding any ability to draw conclusions on initial intervention effectiveness. Third, the adherence to exercise prescription was only based on a self-report of participants. We lacked a objective and scientific means of monitoring the entire program. Fourth, as a feasibility and single-arm study, we failed to control age and disease-related confounding factors, which might have potentially inﬂuenced our observations. Future studies using a randomized controlled intervention protocol and employing app-based, wearable devices are encouraged to expand on this effort.

The observations suggested that our exercise prescription intervention program might promote certain health outcomes, such as cardiovascular function and body composition in middle-aged and older adult Chinese community dwellers. However, we are unable to recommend this program because of the existing limitations. Nonetheless, we recommend that older adults with a range of diseases begin exercise under supervised instruction when initiating training. The benefit is clear, and “start to exercise” should be the top priority for all older adults.

## Appendix

Multimedia Appendix 1 Procedure for measurement with main instruments.

Multimedia Appendix 2 Examples of 3 modes of exercise prescription.

Multimedia Appendix 3 Algorithms for different exercise types.

Multimedia Appendix 4 Changes of health outcomes in the scientific fitness mode (n=80).

## Abbreviations

ABI: ankle/brachial systolic pressure index
ACSM: American College of Sports Medicine
baPWV: brachial-ankle pulse wave velocity
DBP: diastolic blood pressure
IPEPP: intelligent personalized exercise prescription program
MD: mean difference
NCD: noncommunicable chronic disease
SBP: systolic blood pressure
SEVR: subendocardial viability ratio
WHO: World Health Organization
