# Supplementary material for: Intelligent Personalized Exercise Prescription Based on an eHealth Promotion System to Improve Health Outcomes of Middle-Aged and Older Adult Community Dwellers: Pretest–Posttest Study
Source: J Med Internet Res. 2021 May 24;23(5):e28221. doi: 10.2196/28221 (PMC8185615; doi:10.2196/28221)
Supplement: Multimedia Appendix 1 [file jmir_v23i5e28221_app1.docx]

| Internet-based instrument | Parameter | Procedure of measurements |
| --- | --- | --- |
|  |  |  |
| Cardiovascular function monitor  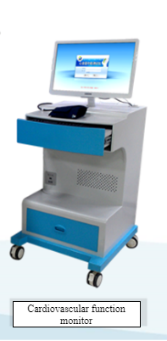 | SBP^a^, DBP^b^, Heart rate, SEVR^c^ | It has a cuff that can be automatically inflated and deflated. A second measurement was automatically performed 3 min after the first one, and the average value was recorded. Before the measurements, participants were required to relax; sit in a chair (feet on the floor with back supported) for >10 min; avoid caffeine, exercise, and smoking for at least 30 min prior; empty their bladder; and remove all clothing covering the location of cuff placement. During the test period participants were instructed to lie on an examination bed and there was no communication between the participant and the observer. |
| Arteriosclerosis detector  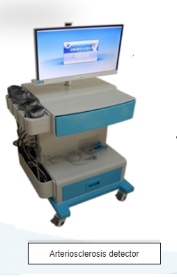 | baPWV^d^ | baPWV (m/s) was measured immediately after cardiovascular function was assessed (with participants instructed to remain supine on the same examination bed without talking). It recorded bilateral brachial and posterior tibial-artery pressure waveforms with an oscillometric method by means of cuffs placed on participants’ arms and ankles. baPWV was calculated automatically for each arterial segment as the path length divided by the corresponding time interval. |
| Body composition monitor  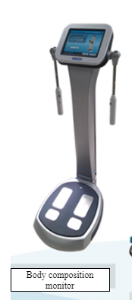 | Weight, BMI, body fat rate, fat-free mass, muscle mass, fat mass | The participants refrained from eating and drinking 3 h before measurements were performed, and were instructed to remove their socks and stand on the machine; electrodes were placed on both hands and feet, and the participants were instructed to lift both arms upright and touch the electrodes with their hands. |
| Bone densitometer  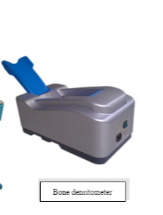 | STI^e^, T value^f^, Z value^g^ | Participants were asked to sit in a chair, take off one of their shoes and socks, and place their heel on the instrument. Both sides of the calcaneus were swabbed with alcohol and coated with coupling agent. Keeping their legs in the same position for approximately 15 seconds, the data were uploaded to the system. |
| Physical fitness detectors |  |  |
| Handgrip strength meter  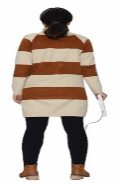 | Handgrip strength, | HGS^h^ (defined here as the maximal HGS of the dominant hand) was measured using a spring-type dynamometer. While standing and extending their arms at their sides, participants were told to squeeze the dynamometer as hard as possible for up to 3 seconds. They were asked to do this three times with 30-second rests between each attempt. |
| Reaction time meter  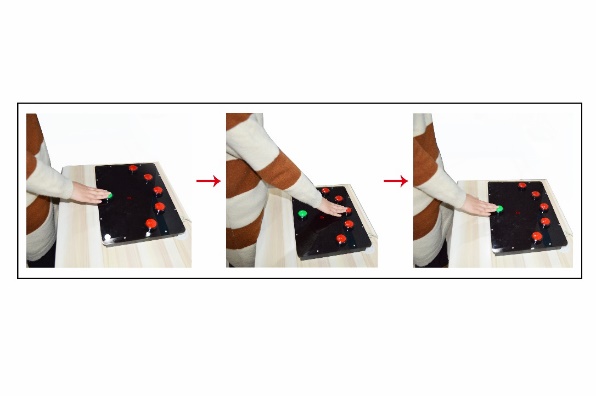 | Agility | During the test, participants were asked to hold down the start button with their middle finger and wait for the signal to be sent. When the light was on, participants needed to press the button as quickly as possible, then wait for the next signal to go off. There were five signals in all. After the participant had completed the fifth response, the mean length of each time of response was calculated. |
| One-leg stand meter with closed eyes  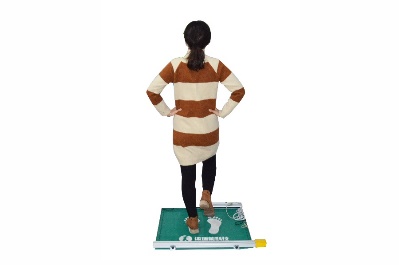 | Balance ability | The individual stands naturally, with the feet in the middle of the test stand, hands on the waist, eyes closed. After stabilizing, the participant was asked to lift one foot when hearing the prompt and the instrument began to record time. |
| Spirometer  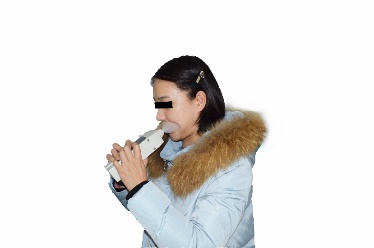 | Vital capacity | The participants inhale deeply until they can no longer breathe, then apply their mouth to the mouth of the spirometer and breathe deeply until they reach the limit. They were asked not to pause or breathe during the test. After two measurements, the spirometer would calculate the maximum value of the test. |
| Flexion measurement instrument of sitting position  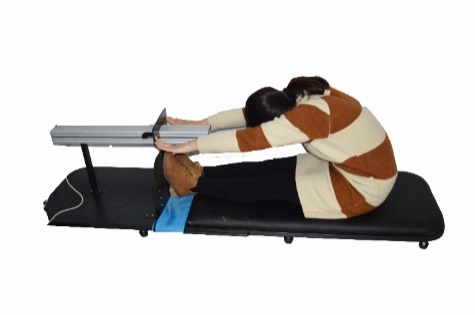 | Flexibility | The participant sits on a mat with legs straight, heels together, toes naturally apart, and the sole of the feet on the tester board; then, palms down, arms together and extended, and upper body flexed, the middle finger tip of both hands is used to push the cursor forward smoothly until they can move no further. After two measurements, the instrument calculates the maximum value of the test. |

^a^SBP: systolic blood pressure.

^b^DBP: diastolic blood pressure.

^c^SEVR: subendocardial viability ratio.

^d^baPWV: brachial-ankle pulse wave velocity.

^e^STI: stiffness index.

^f^T value: was used to evaluate the absolute risk of fracture.

^g^Z value: was primarily used to assess the relative risk of fracture and compared to their peers.

^h^HGS: handgrip strength.
