# Supplementary figures and images for: Intelligent Personalized Exercise Prescription Based on an eHealth Promotion System to Improve Health Outcomes of Middle-Aged and Older Adult Community Dwellers: Pretest–Posttest Study
Source: J Med Internet Res. 2021 May 24;23(5):e28221. doi: 10.2196/28221 (PMC8185615; doi:10.2196/28221)

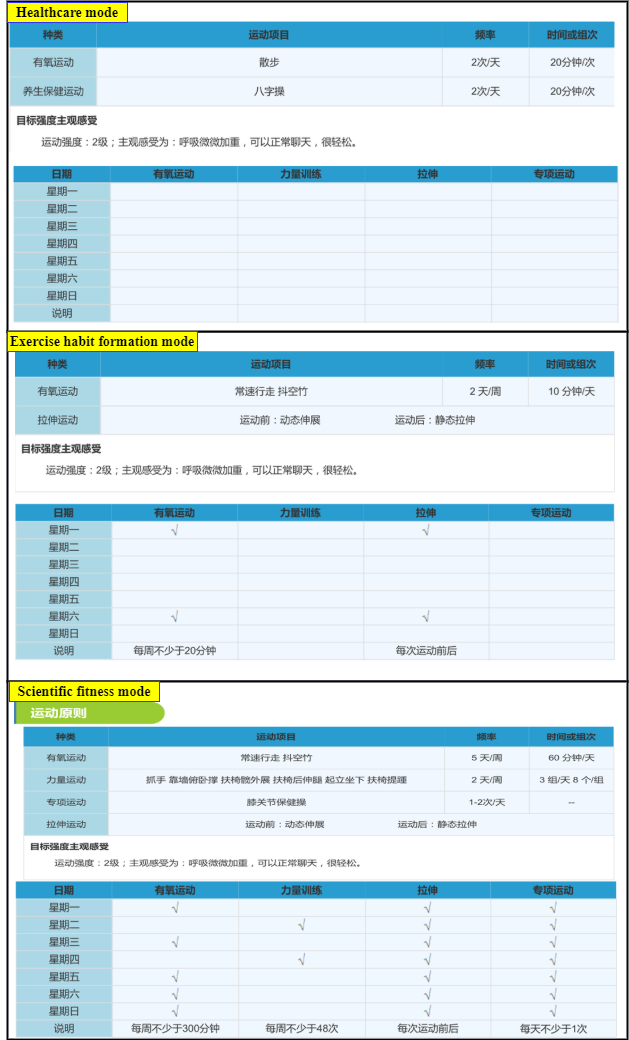

Supplement: Multimedia Appendix 2 [file jmir_v23i5e28221_app2.docx]

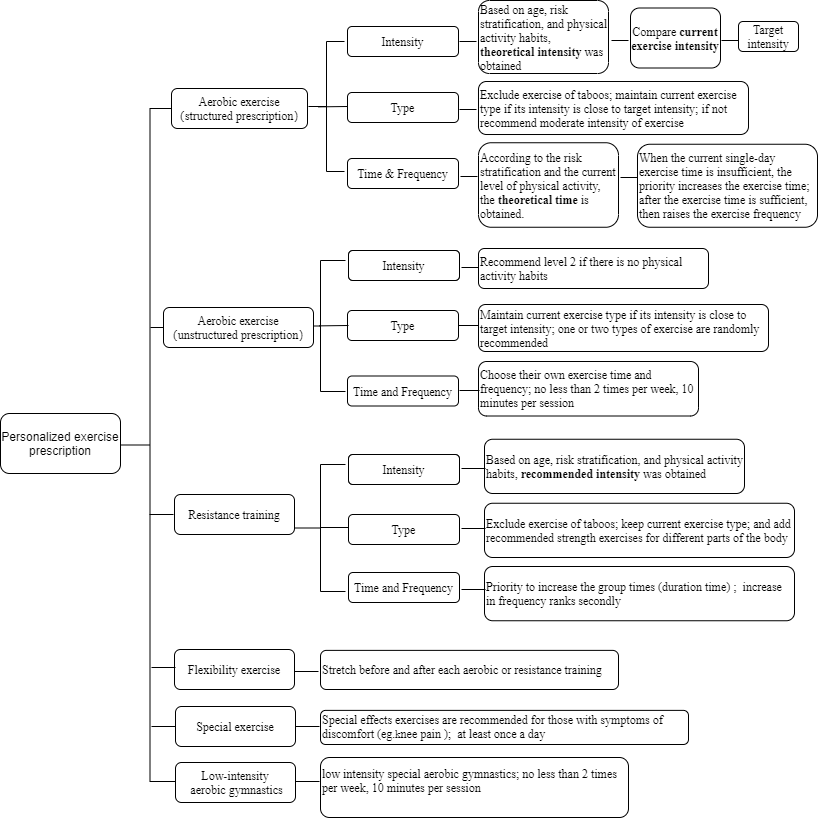

Supplement: Multimedia Appendix 3 [file jmir_v23i5e28221_app3.docx]
