# Supplementary material for: Intelligent Personalized Exercise Prescription Based on an eHealth Promotion System to Improve Health Outcomes of Middle-Aged and Older Adult Community Dwellers: Pretest–Posttest Study
Source: J Med Internet Res. 2021 May 24;23(5):e28221. doi: 10.2196/28221 (PMC8185615; doi:10.2196/28221)
Supplement: Multimedia Appendix 4 [file jmir_v23i5e28221_app4.docx]

|  | Characteristic | Pre, mean (SD) | After, mean (SD) | Mean difference (95% CI) | *P* value |
| --- | --- | --- | --- | --- | --- |
|  |  |  |  |  |  |
| **Cardiovascular function** |  |  |  |  |  |
|  | SBP^a^, mmHg | 125.96 (14.67) | 124.83 (13.86) | 1.13 (−2.25, 4.51) | .153 |
|  | DBP^b^, mmHg | 74.64 (8.73) | 74.15 (9.22) | 0.49 (−1.33, 2.30) | .594 |
|  | Heart rate, bp/min | 68.22 (7.85) | 69.29 (13.18) | −1.08 (−3.58, 1.43) | .395 |
|  | SEVR^c^ | 1.20 (0.20) | 1.23 (0.25) | −0.04 (−0.11, −0.02) | .198 |
|  | baPWV^d^, m/s | 15.70 (2.70) | 16.81 (5.31) | −1.11 (−2.37, −0.15) | .840 |
| **Body composition** |  |  |  |  |  |
|  | Weight, kg | 68.06 (11.80) | 68.52 (11.76) | −0.46 (−1.32, 0.41) | .300 |
|  | BMI, kg/m^2^ | 25.17 (3.64) | 25.90 (3.32) | −0.19 (0.78, 0.40) | .520 |
|  | Body fat rate, % | 26.80 (8.46) | 27.47 (11.90) | −0.28 (−0.84, 0.28) | .325 |
|  | Fat-free mass, kg | 49.85 (9.09) | 50.00 (9.04) | −0.15 (−0.55, 0.26) | .475 |
|  | Muscle mass, kg | 47.19 (8.75) | 46.89 (9.43) | 0.30 (−0.69, 1.30) | .550 |
|  | Fat mass, kg | 18.58 (7.33) | 18.56 (7.21) | 0.03 (−0.64, 0.69) | .939 |
| **Bone mineral density** |  |  |  |  |  |
|  | STI^e^ | 90.63 (23.60) | 90.23 (25.65) | −0.66 (−2.83, 1.51) | .545 |
|  | T value^f^ | −0.82 (1.24) | −0.74 (1.25) | 0.40 (−5.41, 6.21) | .890 |
|  | Z value^g^ | 0.94 (1.88) | 1.60 (4.86) | −0.65 (−1.76, 0.46) | .248 |
| **Physical fitness** |  |  |  |  |  |
|  | Handgrip strength, kg | 26.65 (8.46) | 23.53 (8.34) | 3.11 (2.96, 5.86) | .002 |
|  | Vital capacity, ml | 1947.74 (469.06) | 1696.00 (671.79) | 250.78 (121.63, 379.92) | <.0001 |
|  | Agility, s | 0.76 (0.29) | 1.16 (0.63) | −0.39 (−0.53, −0.26) | <.0001 |
|  | Balance Ability, s | 5.29 (7.56) | 5.23 (7.25) | 0.056 (−2.31, 2.41) | .962 |
|  | Flexibility, cm | 8.21 (6.10) | 9.25 (7.68) | −1.04 (−2.83, 0.75) | .251 |

^a^SBP: systolic blood pressure.

^b^DBP: diastolic blood pressure.

^c^SEVR: subendocardial viability ratio.

^d^baPWV: brachial-ankle pulse wave velocity.

^e^STI: stiffness index.

^f^T value: was used to evaluate the absolute risk of fracture.

^g^Z value: was primarily used to assess the relative risk of fracture and compared to their peers.
